# E-cigarette-/Vape-Associated Lung Injury as a Cause of Interstitial Lung Disease

**DOI:** 10.7759/cureus.58199

**Published:** 2024-04-13

**Authors:** Kathryn B Vess, Nicole Ivan, Joseph Boscia

**Affiliations:** 1 Department of Internal Medicine, Edward Via College of Osteopathic Medicine, Spartanburg, USA; 2 Department of Pulmonary and Critical Care Medicine, Edward Via College of Osteopathic Medicine, Spartanburg, USA

**Keywords:** non-specific interstitial lung disease (nsip), obstructive and restrictive lung diseases, diffuse alveolar, chronic eosinophilic pneumonia, e-cigarette or vaping use-associated lung injury (evali)

## Abstract

E-cigarette-/vape-associated lung injury (EVALI) refers to damage to lung tissue occurring as a result of e-cigarette utilization or via vaping of inhaled nicotine products. Vaping refers to the practice of inhaling an aerosol derived from heating a liquid or gas containing substances such as nicotine, cannabinoids, flavoring, or additives. Battery-operated e-cigarettes or vape pens are the vessels commonly used in this practice. EVALI, first described in the literature in 2019, has a non-specific course, presenting initially with cough and dyspnea. It can progress, however, to interstitial lung disease or result in damage to the lung parenchyma with concomitant inflammation and fibrosis. Imaging findings reflect the development of this inflammation and fibrosis, often visualized as ground-glass opacities on computed tomography (CT) scans. Formal biopsies are not required to make the diagnosis of EVALI, and thus, a gap exists in the scientific literature with regard to the pathology of lungs exposed to non-tetrahydrocannabinol (THC) e-cigarettes. The following case details the clinical course of a 62-year-old male who presented to the outpatient pulmonology office with symptomology and exposure history consistent with EVALI, unique in presentation due to the timeline of his disease development. The patient initially presented to the clinic for the evaluation of a non-productive cough and exertional dyspnea beginning one year ago, with an associated new home oxygen requirement of 2 liters via nasal cannula. The patient's past medical history was relevant for diffuse large B-cell lymphoma treated with the chemotherapeutic regimen that consists of etoposide phosphate, prednisone, vincristine sulfate (Oncovin), cyclophosphamide, doxorubicin hydrochloride (hydroxydaunorubicin), and rituximab, commonly known as EPOCH-R, as well as a social history relevant for a 35-pack-year smoking history. On further questioning, the patient revealed that following cessation of cigarette smoking, he began using non-THC e-cigarettes daily and had been doing so for 10 years prior to symptom onset. Imaging and biopsy findings consisted of a CT of the chest demonstrating concern for interstitial lung disease and an open lung biopsy demonstrating diffuse alveolar damage with eosinophilia. Given the patient's history, clinical symptoms, and imaging findings, a diagnosis of EVALI was established. This case was documented not only to increase awareness of the rising incidence of EVALI as the use of e-cigarettes and vapes becomes increasingly popular but also to further understand the inhalational injury sustained from non-THC e-cigarettes and other inhalational practices.

## Introduction

Interstitial lung disease (ILD) refers to a diverse group of lung diseases which cause damage to the alveoli, bronchioles, and series of connective ducts [1]. Inflammation in the airways over time leads to airway remodeling and deposition of connective tissue via fibroblasts ultimately causing fibrosis [2]. Symptomology of ILD at initial presentation is often non-specific, consisting mostly of chronic, non-productive cough and persistent exertional dyspnea, making the determination of an underlying etiology very difficult.

ILD can have a variety of underlying etiologies most commonly belonging to one of three groups: exposure, autoimmune, and idiopathic. Exposures related to ILD include silica, asbestos, nicotine, beryllium, radiation, and medications such as antiarrhythmics, antineoplastics, antibiotics, and anti-inflammatory drugs [3-5].

Cigarette smoking is the most notorious of exposures related to the development of ILD due to the toxins present in cigarette smoke [6-8]. As nicotine and tobacco use has shifted to include the use of e-cigarette devices, the nature of the toxin exposure has changed as well. E-cigarettes and vapes represent a unique toxin exposure due to both the broad range of nicotine-containing liquids being inhaled and the composition of the atomizers from which they are being smoked. The atomizers of e-cigarette devices themselves have been found to contain heavy metals, and when combined with the thousands of different solvents available on the market, innumerable combinations of toxins are created that are then being inhaled [9]. Flavoring additives used in vape pens additionally contribute to the overall toxin exposure to users.

The health repercussions of exposure to many of these toxins are only beginning to be understood. For instance, diacetyl and 2,3-pentanediol have been shown to disrupt the cilia of bronchial epithelial cells causing damage to the mucociliary escalator. This results in difficulty clearing mucus and pathogens from the respiratory system and has also been linked to cases of bronchiolitis [10-12]. When vegetable glycerin, a substance commonly used in vape liquids, and propylene glycol decompose, harmful compounds are generated, such as formaldehyde, acrolein, and acetaldehyde. These compounds subsequently cause oxidative stress resulting in inflammation, airway injury, and remodeling [13].

The vape system itself is comprised of a chamber and a heating element [12]. The solvent is dispensed into the chamber, and the coiled metal heating element warms the liquid to 4000 times its initial temperature to reach the gaseous state [12,14]. The continual heating and cooling of the metal heating element as the system is used causes the vaporization of the trace metals found in the device resulting in the mobilization of small, toxic particles which are then inhaled [12]. Particles smaller than 1-2 micrometers are able to reach the terminal airways and cause widespread parenchymal damage [15]. This widespread parenchymal damage and overall insult to the lungs secondary to the both use of these systems and exposure to these noxious aerosolized compounds is referred to as e-cigarette-/vape-associated lung injury (EVALI).

EVALI describes any pathologic injury to the lungs that happens in association with exposure to e-cigarettes or vaping [16]. Documented outcomes of EVALI have included a broad spectrum of diseases from asymptomatic patients with incidental radiologic findings to those with lipoid pneumonia and diffuse alveolar hemorrhage [16]. EVALI remains a diagnosis of exclusion largely based on patient history of exposure to e-cigarettes, computed tomography (CT) imaging, and often bronchiolar lavage [16].

Radiologic findings of EVALI on CT imaging most commonly show non-specific bilateral ground-glass opacities [16]. Bronchiolar lavage, though not necessary for diagnosis, demonstrates eosinophilic predominance of the tissues with cytology studies demonstrating the presence of lipid-laden macrophages [16-18]. As biopsies are also not routinely performed to confirm the diagnosis of EVALI much like bronchoalveolar lavage, several studies have included them in the workup and revealed the most common histopathological findings to be diffuse alveolar damage, foamy macrophages, and interstitial pneumonitis [16].

## Case presentation

A 62-year-old male presented to the pulmonology outpatient office for the evaluation of worsening shortness of breath that began one and a half years ago. The patient described his shortness of breath as feeling "unable to fully inhale" and reported worsening dyspnea with activity. The patient also noted a new supplemental oxygen requirement of 2 liters and associated non-productive cough. He reported that despite oxygen therapy, he had not experienced any improvement in his symptoms. The patient's past medical history was significant for diffuse large B-cell lymphoma which was treated with six cycles of chemotherapy with etoposide phosphate, prednisone, vincristine sulfate (Oncovin), cyclophosphamide, doxorubicin hydrochloride (hydroxydaunorubicin), and rituximab, commonly known as EPOCH-R, four years prior. The patient's social history was also relevant for a 35-pack-year history of smoking cigarettes wherein a standard pack of cigarettes contains 20 cigarettes. He reported quitting approximately 10 years ago; however, in its place, he began using e-cigarettes and had continued doing so until the present day.

Pulmonary function tests previously conducted by the patient's former pulmonologist showed reduced forced expiratory volume (FEV1), forced vital capacity (FVC), and FEV1/FVC ratio, as well as a reduced total lung capacity (TLC), findings concerning for mixed obstructive and restrictive lung disease. The patient also had a series of CT scans of the chest beginning three years prior to presentation, each showing non-specific, yet concerning, findings for ILD. One year prior to presenting at the office, the patient also had a bronchoscopy with bronchoalveolar lavage performed which revealed grossly hyperemic airways but was negative for malignancy and culture negative for any infectious processes. 

**Figure 1:**
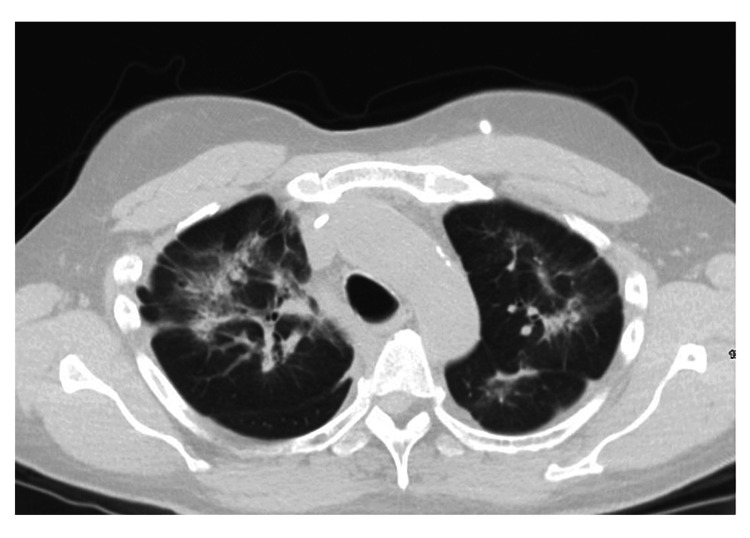
CT of the chest demonstrating bilateral interstitial opacities

After the evaluation of the patient in the clinic, the patient was sent for a repeat CT scan which showed diffuse interstitial opacities without honeycombing as seen in Figure 1.

**Figure 2:**
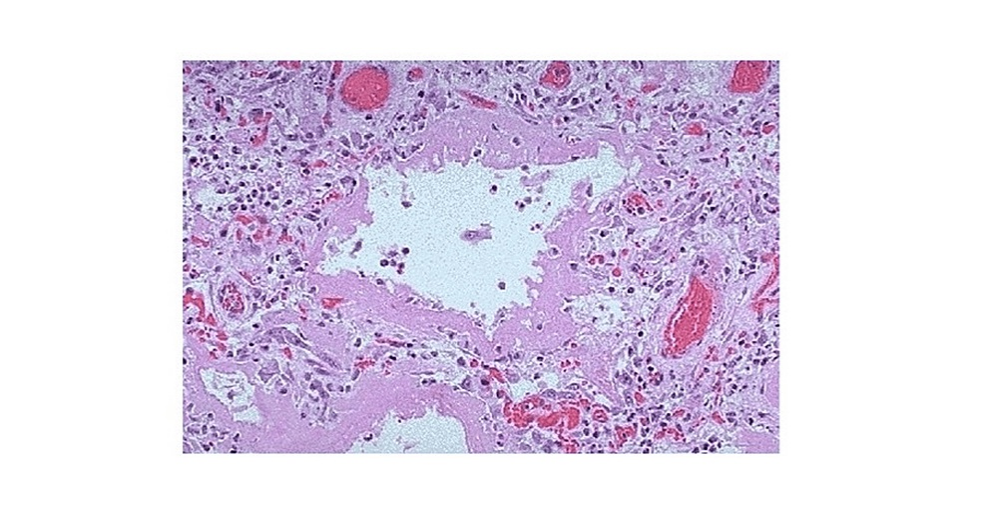
Representative pathology samples of the right lung demonstrating diffuse alveolar damage with eosinophilia Source: https://webpath.med.utah.edu/LUNGHTML/LUNG133.html

Given the non-specific nature of the CT findings and in the context of the previous workup, the patient was sent for an open lung biopsy. A right lung wedge biopsy was performed, and the tissue was sampled from the patient's upper, middle, and lower lobes. These tissue samples were reviewed by both a pathologist at the home institution and a specialist in pulmonary pathology. These samples showed alveolar damage with eosinophilia, a representation of which can be seen in Figure 2.

These findings in combination with the CT imaging and patient history were consistent with the diagnosis of alveolar damage secondary to EVALI.

The patient was then hospitalized during which time he received five weeks of 30 mg intravenous methylprednisolone. He experienced a relatively benign hospital course and was discharged home on his baseline home oxygen requirement of 2 liters via nasal cannula. The patient reported improvement in symptoms with regard to his dyspnea and cough and continued daily treatment with single-inhaler triple therapy (SITT) following discharge.

## Discussion

Inhalation injuries can damage the airways, lung parenchyma, or both [17]. The ability of a substance to damage the lung parenchyma is determined by the relative lipid solubility of a substance [17]. A more lipid-soluble substance will bypass the airways and selectively induce damage in the parenchyma [17]. As the majority of commercially available e-cigarette solvents are mixed into a base of vegetable glycerin, a highly lipid-soluble substance, inflammatory substances and solvents are more likely to reach the lung parenchyma and induce damage. The inflammatory solvents that do reach the lung parenchyma elicit inflammation of the epithelium and of the endothelium of the pulmonary capillaries which leads to collagen deposition and capillary leakage, respectively [17]. Widespread endothelial damage by these substances can be significant enough to cause acute respiratory distress syndrome; however, this reaction is rare [17].

Diffuse alveolar damage secondary to inflammation of the parenchymal epithelium is the most commonly seen pathology associated with EVALI [17]. The toxic insult results in edematous changes, epithelial cell death, and sloughing of multiple cell layers, and if the toxic inhalant quantity is significant and sustained over an extended period, this inflammation can lead to the formation of hyaline membranes and fibroblast proliferative changes [17].

Damage induced to the lung parenchyma secondary to EVALI is comparable to the damage elicited by traditional inhalation lung insults [18]. However, due to the highly water-soluble nature of the chemicals found in common combustion reactions, airway injuries tend to be proximal involving the airway and tend to spare the lung parenchyma [18]. Damage to the airways as opposed to the parenchyma is in part due to pulmonary anatomy as the majority of heat is dispelled prior to passing the vocal cords via the oropharynx and nasopharynx [17,18]. However, similar to EVALI, if lipid-soluble toxins are present in the combustible materials, lower airway or parenchymal damage can occur along with upper airway disease.

Interesting features of this case include the histological finding of diffuse alveolar damage accompanied by eosinophilia, which can be suggestive of eosinophilic pneumonia. Many cases of acute-onset eosinophilic pneumonia have been observed following exposure to vaping or e-cigarette usage. However, in each of these cases, the duration of time between e-cigarette use and symptom onset was a few weeks. In this case, the patient had almost a decade of exposure before he became symptomatic [4,19]. Another interesting feature is the patient's delayed development of eosinophilia as prior bronchoscopy with bronchoalveolar lavage did not reveal this finding.

Patients with EVALI typically respond well to treatment with corticosteroids as well as cessation of e-cigarette use [19]. No standardized treatment dose exists; however, a retrospective study of patients hospitalized for EVALI showed that patients had marked improvement with one month of treatment with methylprednisolone [13]. Outcomes were varied following treatment, ranging from complete resolution of symptoms and imaging pathology to persistent pulmonary ILD and an associated persistence of symptoms [19]. 

## Conclusions

EVALI is associated with increasing cases of ILD. Clinicians must be diligent in taking accurate social histories of their patients to evaluate for smoking/vaping, especially in patients with pulmonary concerns. EVALI symptoms are non-specific, and radiologic findings can be attributed to a variety of etiologies, causing the diagnosis to be easily missed. Treatment is effective with a short course of corticosteroids and e-cigarette smoking cessation, but due to the novelty of this disease, new treatment options are constantly being considered. Patients are likely to make a full recovery, as seen in this case, with the patient returning home on his home oxygen requirement and maintenance of remission with the use of SITT. If not diagnosed in a timely manner, however, EVALI can potentially lead to irreversible lung injury. 
